# Theoretical Aspects of Resting-State Cardiomyocyte Communication for Multi-Nodal Nano-Actuator Pacemakers

**DOI:** 10.3390/s20102792

**Published:** 2020-05-14

**Authors:** Pengfei Lu, Mladen Veletić, Jacob Bergsland, Ilangko Balasingham

**Affiliations:** 1 The Intervention Centre, Oslo University Hospital, 0372 Oslo, Norway; veleticm@gmail.com (M.V.); jacobbergsland622@gmail.com (J.B.); ilangko.balasingham@medisin.uio.no (I.B.); 2 Computer College, Weinan Normal University, Weinan 714099, China; 3 Faculty of Medicine, University of Oslo, 0315 Oslo, Norway; 4 Faculty of Electrical Engineering, University of Banja Luka, 78000 Banja Luka, Bosnia and Herzegovina; 5 Department of Electronic Systems, Norwegian University of Science and Technology, 7491 Trondheim, Norway

**Keywords:** body area network, cardiomyocytes, cellular communication, intra-body communication, molecular communications

## Abstract

The heart consists of billions of cardiac muscle cells—cardiomyocytes—that work in a coordinated fashion to supply oxygen and nutrients to the body. Inter-connected specialized cardiomyocytes form signaling channels through which the electrical signals are propagated throughout the heart, controlling the heart’s beat to beat function of the other cardiac cells. In this paper, we study to what extent it is possible to use ordinary cardiomyocytes as communication channels between components of a recently proposed multi-nodal leadless pacemaker, to transmit data encoded in subthreshold membrane potentials. We analyze signal propagation in the cardiac infrastructure considering noise in the communication channel by performing numerical simulations based on the Luo-Rudy computational model. The Luo-Rudy model is an action potential model but describes the potential changes with time including membrane potential and action potential stages, separated by the thresholding mechanism. Demonstrating system performance, we show that cardiomyocytes can be used to establish an artificial communication system where data are reliably transmitted between 10 s of cells. The proposed subthreshold cardiac communication lays the foundation for a new intra-cardiac communication technique.

## 1. Introduction

**Figure 1:**
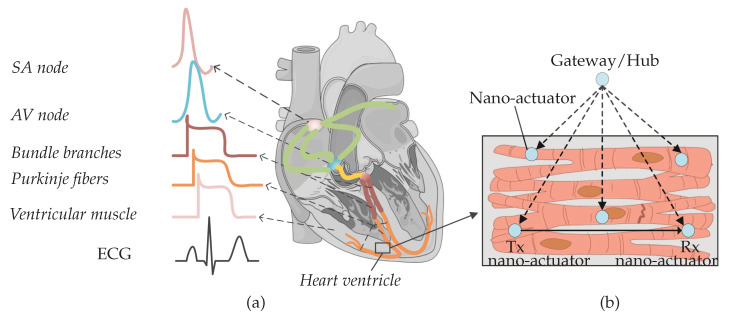
(**a**) Nodes from different parts of the heart produce diverse action potential signals. The composition of action potentials generates an ECG signal. (**b**) Nano-actuator pacemaker network in the heart ventricle: multiple nano-actuators are distributed in the ventricle and are coordinated by the gateway/hub. The nano-actuators are envisioned to share information to enhance their abilities [1]. The figure is adapted from an existing image provided by Servier Medical Art by Servier, licensed under a Creative Commons Attribution 3.0 Unported License.

The heart’s function is dependent on cardiomyocytes contracting in a coordinated fashion when electrically stimulated by the conduction system (Figure 1a). The electrical activity starts at the Sinoatrial (SA) node—a node of specialized cardiomyocytes that initiates a synchronized electrical impulse. The SA node is a natural pacemaker and the electrical activity spreads to the right and left atria, depolarizing them to contract. The impulse spreads to the ventricles through the Atrioventricular (AV) node, the right bundle branches (RBBs) and left bundle branches (LBBs), and the Purkinje fibers. Electrocardiogram (ECG) is used to record cardiac electrical activity as a combination of all action potentials generated by the nodes and the cardiomyocytes.

In the presence of heart muscle damage, heart conduction may be disturbed, and artificial pacemakers are needed to re-establish regular cardiac operation [1,2]. In our recent paper, we discussed state-of-the-art pacemakers, and proposed a conceptual nano-actuator-network-based leadless pacemaker to overcome limitations imposed by battery longevity [1] (Figure 1b). Such a leadless device would pace numerous parts of the heart, using nano-actuators inter-connecting with individual cardiomyocytes, perform basic stimulation tasks by injecting current to the cytosol, and work in synchrony to optimize the energy used by individual batteries in the devices. Evoked electrical impulses/action potentials from actuated cardiomyocytes could then coordinate contraction throughout the heart muscle and lead to a normal heartbeat.

The nano-actuators have limited functionality unless they communicate with each other and coordinate pacing activities within the network. An example of the required information that needs to be communicated by a nano-actuator node includes notification of a mistiming in the contraction of the ventricles, which is a sign to other nodes to pace. Integrating the communications paradigm with the proposed network provides an energy-efficient method of pacing and may enable clinical patient management using a gateway/nano-hub [1,3]. The nano-hub primarily coordinates the nodes (as shown in Figure 1b) but can, in addition, send collected heart information to an external receiver.

Two nanoscale communications options have recently been proposed for the study of short-range communication between nano-transceivers [4,5,6,7]: *electromagnetic nano-communications* and *molecular communications* [7,8]. The terahertz band (0.1–10 THz) is envisioned to be used in wireless electromagnetic nanotechnology. The terahertz band addresses the increasing demand in classical networking domains by alleviating spectrum scarcity and capacity limitations of current wireless systems [9,10]. This frequency domain is less explored for communication, compared to frequency regions below and above this band, i.e., microwaves and far infra-red [11,12]. Though such terahertz bands can enable communication between nanoscale entities, a practical implementation for intra-body use is challenging due to constraints such as antenna size and wavelength of the electromagnetic signal, power consumption, and dampening in saltwater environments, such as the body.

Conversely, molecular communication has emerged as a promising networking methodology in intra-body nano-networks due to the dimensional similarities with biological structures [13,14,15,16]. Molecular communication can be engineered in two ways: an entirely artificial device could be designed for communication using signaling ions or molecules, or the molecular communication capabilities which occur ubiquitously at all levels of biological systems including ion, molecule, cell, tissue, and organ levels could be engineered to transmit data [17,18,19].

**Figure 2:**
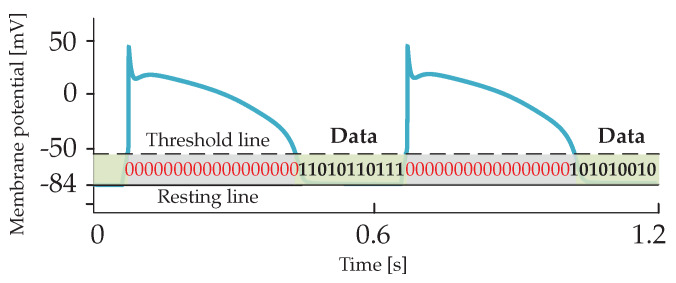
Encoding subthreshold membrane potentials are envisioned to be transmitted over cardiac cellular infrastructure within time bins between consecutive action potentials.

In this work, we explore the latter approach and use of membrane potential perturbations generated and propagated by cardiomyocytes when external stimuli and/or ion exchange occurs between the intra- and extra-cellular environments. In the considered scenario, if a stimulus applied to a targeted cardiomyocyte by a nano-actuator paces the heart, the cardiomyocyte should respond with *action potentials*. The action potential occurs when the membrane potential reaches the specified threshold values (typically −60 mV). Conversely, if a stimulus applied to the targeted cardiomyocyte by the nano-actuator is used to transmit data to another nano-actuator(s), the cardiomyocyte should respond by creating *subthreshold membrane potentials*. *The subthreshold membrane potential thus refers to the membrane potential whose peak amplitude is below the specified action potential threshold.* We envision the utilization of subthreshold membrane potentials in the time period between consecutive action potentials and use them as encoding signals (Figure 2). In other words, transmission happens during the ventricular diastole phase. Of note, the theoretical framework presented in the following applies to different types of cardiomyocytes, e.g., those originating from the ventricles or atria. Nonetheless, by adopting a set of cell-specific parameters for the numerical simulations, we present numerical results that applies to the ventricular cardiomyocytes only.

Cardiac infrastructure indeed developed during evolution to conduct the signaling messages among cardiomyocytes and coordinate heartbeats. By utilizing encoding of subthreshold membrane potentials, the cardiac signaling system may be transformed into a more advanced cardiac communication system. We refer to the proposed communications paradigm between nano-actuators as *Subthreshold Cardiac Communication.* In the work presented, we assume that the encoding subthreshold membrane potentials does not interfere with action potentials, nor affect normal heart function.

The rest of the paper is organized as follows. In Section 2, we propose a basic cardiac communication system and to linearize the cardiac cell membrane circuit by using the quasi-active method. In Section 3, we characterize the impacts of various noise sources on system performance. In Section 4, we provide numerical results. Finally, in Section 5, we discuss and conclude the study.

## 2. Subthreshold Cardiac Communication System

We considered the communication system between a transmitting nano-actuator and receiving nano-actuators within the multi-nodal pacemaker network. A small membrane patch connected to the transmitting nano-actuator is located in a selected compartment of the emitting cardiomyocyte and is used for stimulation. The emitting cardiomyocyte can respond to the provided simulation patterns with action potentials or subthreshold membrane potentials, which are both distributed forward through the unidirectional propagation channel [20,21]. The signaling/communication channel consists of cardiomyocytes connected by specialized gap-junction-like channels [22]. Gap junctions can be observed as aggregations of single dynamic and multi-functional channels, called connexins, which play a complicated and essential role in the entire conduction system of the heart [23]. A small membrane patch connected to the receiving nano-actuator is located in a selected compartment of the receiving cardiomyocyte. The receiving cardiomyocyte responds either with action potentials or subthreshold membrane potentials to the propagated signals. In this scenario, we inspect the performance of the described system when the transmitting nano-actuator sends encoded data during the subthreshold time-period by generating a stimulus to the emitting cardiomyocyte. Table 1 defines symbols used throughout the paper.

We opt to use one-dimensional cable theory to analyze the propagation of unidirectional subthreshold membrane potentials along cylindrically shaped cardiomyocytes [24,25]. The one-dimensional cable theory is widely used in the literature to model excitable tissues, e.g., nerve axons and skeletal muscle fibers [25]. Although cardiomyocytes generally form a strand consisting of individual cells with irregular shapes, the theory could potentially lead to inaccurate numerical results. Nonetheless, as this work lays the foundation for a new concept of biological communication paradigm, we believe that applying the one-dimensional cable theory reduces the complexity in this initial study. Besides, we use the quasi-active method to linearize the membrane’s active channel kinetics into phenomenological impedances when subthreshold membrane potentials have small variations around the holding potential (The value of the holding potential refers to a specific value used as the baseline to determine fluctuations of the membrane potential.) [26,27,28,29]. The phenomenological impedance can have positive or negative components, depending on the difference between the holding potential and the reversal potential of different ionic channels [26]. The linearization is exclusively valid in the considered subthreshold regime where non-linearities, encountered in the creation of action potentials, are not expected to occur. The linearization, however, ensures us to use tools for studying linear systems and analyze the behaviour of this complex biological system.

### 2.1. Transfer Impedance

According to the one-dimensional cable theory, the propagation channel is equivalent to the cable that consists of the *membrane impedance* (per unit length), the *intracellular impedance*, and the *gap junction impedance*.

**Figure 3:**
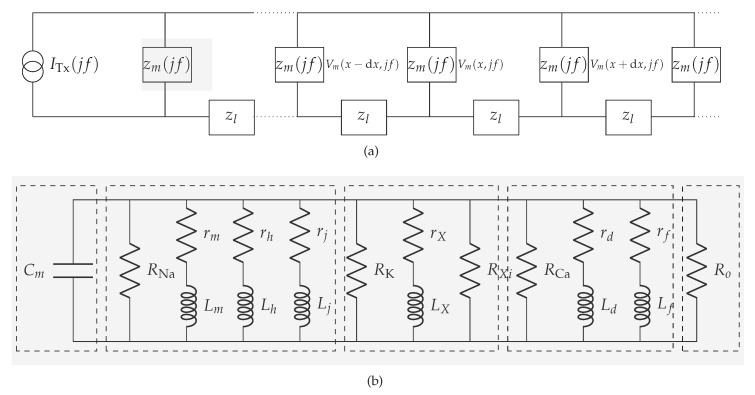
Communication channel in the subthreshold cardiac communication system. (**a**) The general representation of the subthreshold cardiac communication channel as a one-dimensional cable. (**b**) The linearized membrane circuit corresponding to the shaded block in Figure 3a. (1)-segment consists of the capacitor derived from the bilayer membrane; (2)-segment consists of passive components derived from the voltage-gated sodium channels; (3)-segment consists of passive components derived from the voltage-gated potassium channels; (4)-segment consists of passive components derived from the voltage-gated slow inward current which mainly contains calcium channels; (5)-segment consists of the resistor derived from the plateau potassium current and background current.

We denote the membrane impedance as $z_{m}$, as shown in Figure 3a, and define using the resistivity of the membrane $Z_{m}$

(1) $$\begin{array}{c} z_{m}\left(jf\right)=\frac{Z_{m}\left(jf\right)}{2\pi a}, \end{array}$$

where *a* denotes the radius of the cardiomyocyte strand. Aiming to define $Z_{m}$, we first inspect the transmembrane current components on a membrane patch (i.e., the currents that depend not only on membrane potentials but also on opening/closing of ionic channels, e.g., sodium, potassium, and calcium, and the currents that depend solely on membrane potentials), and then apply the quasi-active method [26,27,29]. The linearized circuit for a membrane patch in a specific holding potential in the subthreshold regime ($\left[-84,-60\right]$ mV) is shown in Figure 3b. Detailed linearization method used to define the circuit in Figure 3b is shown in Appendix A.1. Following Figure 3b, we now define $Y_{m}$

(2) $$\begin{array}{ccc} Y_{m}\left(jf\right) & = & G_{c}+j2\pi fC_{m}+\frac{1}{r_{m}+j2\pi fL_{m}}+\\ & & \frac{1}{r_{h}+j2\pi fL_{h}}+\frac{1}{r_{j}+j2\pi fL_{j}}+\\ & & \frac{1}{r_{X}+j2\pi fL_{X}}+\frac{1}{r_{d}+j2\pi fL_{d}}+\frac{1}{r_{f}+j2\pi fL_{f}}, \end{array}$$

where $G_{c}=G_{\mathrm{Na}}+G_{K}+|G_{Xi}+G_{\mathrm{Ca}}+G_{o}$, and $G_{\mathrm{Na}}$, $G_{K}$, $G_{Xi}$, $G_{\mathrm{Ca}}$ and $G_{o}$ are reciprocals of $R_{\mathrm{Na}}$, $R_{K}$, $R_{Xi}$, $R_{\mathrm{Ca}}$ and $R_{o}$, respectively. Finally,

(3) $$\begin{array}{c} Z_{m}\left(jf\right)=\frac{1}{Y_{m}\left(jf\right)}. \end{array}$$

The intracellular impedance and the gap junction impedance are commonly referred to as the equivalent longitudinal impedance (per unit area) [30,31]. We denote the equivalent longitudinal impedance as $z_{l}$ (Figure 3b), and define using the equivalent longitudinal resistivity $Z_{l}$ [24]

(4) $$\begin{array}{c} z_{l}=\frac{Z_{l}}{\pi a^{2}}, \end{array}$$

where the typical value of $Z_{l}$ ranges from 0.6 to 36.6 kΩ· cm [24].

The equivalent impedance of the overall propagation channel, hereinafter referred to as the *transfer impedance,* finally stems from the one-dimensional cable equation [29]

(5) $$\begin{array}{c} \frac{\partial^{2}V_{m}\left(x,jf\right)}{\partial x^{2}}=\gamma^{2}\left(jf\right)V_{m}\left(x,jf\right). \end{array}$$

Equation (5) characterizes the membrane potential dynamics in the frequency domain at different propagation distances, where γ(jf) is the propagation constant expressed as [24,27]

(6) $$\begin{array}{c} \gamma \left(jf\right)=\sqrt{\frac{z_{l}aS_{V}}{2z_{m}\left(jf\right)}}, \end{array}$$

where $S_{V}$ is the surface-to-volume ratio of the cardiomyocyte. With the propagation distance set to infinity as the boundary condition, i.e., V(∞,jf)=0, we define the transfer impedance of the channel [29]

(7) $$\begin{array}{c} Z\left(x,jf\right)=\sqrt{\frac{z_{m}\left(jf\right)z_{l}}{2aS_{V}}}\exp\left[-x\sqrt{\frac{z_{l}aS_{V}}{2z_{m}\left(jf\right)}}\right]. \end{array}$$

### 2.2. Noiseless Input-Output Relation

Without any loss of generality, we consider the unipolar non-return-to-zero (NRZ) line code as the stimulus applied to the emitting cardiomyocyte and data to be communicated from one nano-actuator to another. Aiming to ease the formulation of a complete analytical framework, we characterize the NRZ line code in the frequency domain defining its Power Spectral Density (PSD) as

(8) $$\begin{array}{c} S_{Tx}\left(jf\right)=\frac{A^{2}T_{s}}{4}sinc^{2}\left(jfT_{s}\right)+\frac{A^{2}}{4}\delta \left(jf\right), \end{array}$$

where *A* is the applied current amplitude denoting transmission of bit 1 of duration $T_{s}$, *f* is the operating frequency, and δ is the Dirac delta function. *A* = 0 denotes transmission of bit 0.

Referring to (7) and (8), the output voltage PSD in the receiving cardiomyocyte is defined as

(9) $$\begin{array}{c} S_{Rx}\left(jf\right)=|Z\left(x,jf\right){|}^{2}S_{Tx}\left(jf\right). \end{array}$$

## 3. Noise in the Subthreshold Cardiac Communication System

**Figure 4:**
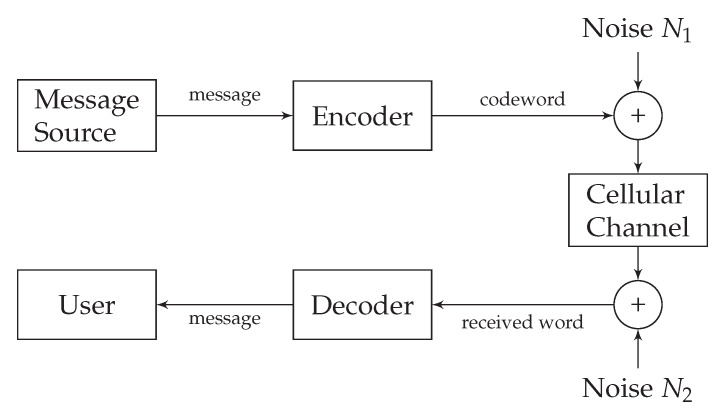
Noisy subthreshold cardiac communication channel model.

Field stimulation and direct stimulation are the two approaches used for the stimulation of cardiomyocytes. With field stimulation, the microelectrode is not directly fixed to the cell. The stimulation affects the membrane through the extracellular solution, which leads to the generation of membrane potential fluctuations [32]. With direct stimulation–an approach used by the recently proposed nano-actuators [1]-the microelectrode is attached to the cardiomyocyte directly. This approach, however, induces the environmental disturbance in the form of input-dependent noise [33,34], and the membrane-related noise [35]. We refer to the input-dependent noise as the encoding noise. Encoding- and membrane-related noise are denoted as $N_{1}$ and $N_{2}$ in the cardiomyocyte communication system as shown in Figure 4.

### 3.1. Encoding Noise

With the effect of encoding noise reflected through $i_{N_{1}}\left(t\right)$, the injected signal/current is

(10) $$\begin{array}{c} {\tilde{i}}_{Tx}\left(t\right)=i_{Tx}\left(t\right)+i_{N_{1}}\left(t\right), \end{array}$$

where $i_{Tx}\left(t\right)$ denotes the noiseless component. $i_{N_{1}}\left(t\right)$ has already been studied in the relevant literature and derived from an autoregressive random process w(t) as [34]

(11) $$\begin{array}{c} i_{N_{1}}\left(t\right)=i_{Tx}\left(t\right)⊛w\left(t\right), \end{array}$$

where ⊛ denotes convolution. In a complex z-domain, w(t) is discretized as $W\left(z\right)=a_{0}+a_{1}z^{-1}+a_{2}z^{-2}+a_{3}z^{-3}+\cdots +a_{n-1}z^{-n+1}$, where $a_{0}$, $a_{1}$, $a_{2}$⋯$a_{\left(n-1\right)}$ are the coefficients of the *n*-th order autoregressive random process. Considering W(z) as a linear filter and replacing *z* with $e^{j2\pi f}$, we define the PSD of the encoding noise $N_{1}$

(12) $$\begin{array}{c} S_{N_{1}}\left(jf\right)=|W\left(jf\right){|}^{2}S_{Tx}\left(jf\right). \end{array}$$

Finally, the PSD of the input affected by the encoding noise is

(13) $$\begin{array}{c} {\tilde{S}}_{Tx}\left(jf\right)=S_{Tx}\left(jf\right)+S_{N_{1}}\left(jf\right). \end{array}$$

### 3.2. Membrane-Related Noise

Compared to encoding noise, the membrane-related noise is more complex and composed of the (1) *voltage-gated channel noise* induced by stochastic opening/closing of the voltage-gated channels, (2) *shot noise* induced by random ionic release, and (3) *thermal noise* induced by intrinsic circuit dynamics [36,37].

**Figure 5:**
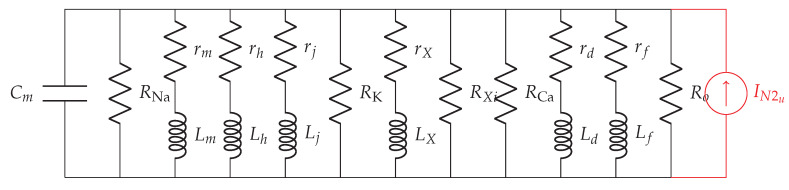
The linearized noisy membrane circuit.

We opt to describe membrane-related noise following the rationale presented in [37,38,39], where the subthreshold neuronal membrane potential noise was characterized. Owing to the similar excitable properties of neurons and cardiomyocytes, we represent the membrane-related noise source as an equivalent Gaussian current source and denote with $I_{N2_{u}}$ in Figure 5. In the following, we characterize membrane-related noise per unit distance assuming that ionic channels are homogeneously spread over the cellular membrane.

#### 3.2.1. Voltage-Gated Channel Noise

We model the effect of stochastic opening/closing of voltage-gated channels in unit distance via conductance variations by defining a noise current $i_{n_{2}}^{1}$ in μA/cm

(14) $$\begin{array}{ccc} i_{n_{2}}^{1}\left(t\right) & = & {\tilde{i}}_{K}\left(t\right)+{\tilde{i}}_{\mathrm{Na}}\left(t\right)+{\tilde{i}}_{\mathrm{Ca}}\left(t\right)\\ & = & {\tilde{g}}_{K}\left(t\right)\left(E_{K}-V_{m}^{0}\right)+{\tilde{g}}_{\mathrm{Na}}\left(t\right)\left(E_{\mathrm{Na}}-V_{m}^{0}\right)+{\tilde{g}}_{\mathrm{Ca}}\left(t\right)\left(E_{\mathrm{si}}-V_{m}^{0}\right), \end{array}$$

where ${\tilde{i}}_{K}\left(t\right)$, ${\tilde{i}}_{\mathrm{Na}}\left(t\right)$ and ${\tilde{i}}_{\mathrm{Ca}}\left(t\right)$ are the noisy currents produced by the random opening/closing of potassium, sodium and calcium channels, respectively. ${\tilde{g}}_{K}\left(t\right)$, ${\tilde{g}}_{\mathrm{Na}}\left(t\right)$ and ${\tilde{g}}_{\mathrm{Ca}}\left(t\right)$ denote conductance variations of respecting ionic channels around their steady-state values when the holding potential is $V_{m}^{0}$. $E_{K}$, $E_{\mathrm{Na}}$ and $E_{si}$ are the reversal potential of potassium, sodium and calcium, respectively. The conductance variation is a collective phenomenon of multiple channels, not a single channel, and depends on the length of the propagation channel and ionic channel density [40,41]. Due to the tiresome mathematical derivation, we derive the PSD of the corresponding ionic current components (${\tilde{S}}_{K}\left(jf\right)$, ${\tilde{S}}_{\mathrm{Na}}\left(jf\right)$ and ${\tilde{S}}_{\mathrm{Ca}}\left(jf\right))$ in Appendix A.2. The PSD of the voltage-gated channel noise is then

(15) $$\begin{array}{c} {\tilde{S}}_{N_{2}}^{1}\left(jf\right)={\tilde{S}}_{K}\left(jf\right)+{\tilde{S}}_{\mathrm{Na}}\left(jf\right)+{\tilde{S}}_{\mathrm{Ca}}\left(jf\right). \end{array}$$

#### 3.2.2. Shot Noise

The shot noise is affected by the random ionic release when different types of cations depolarize the cellular membrane and generate the membrane fluctuation while propagating to the cytosol. We adapt the PSD of the shot noise from the Schottky’s formula and define [36]

(16) $$\begin{array}{c} {\tilde{S}}_{N_{2}}^{2}\left(jf\right)=2\left(q_{K}{\tilde{I}}_{K}\left(jf\right)+q_{\mathrm{Na}}{\tilde{I}}_{\mathrm{Na}}\left(jf\right)+q_{\mathrm{Ca}}{\tilde{I}}_{\mathrm{Ca}}\left(jf\right)\right), \end{array}$$

where $q_{K}$, $q_{\mathrm{Na}}$ and $q_{\mathrm{Ca}}$ are the charges of the moving potassium, sodium and calcium particles, respectively. ${\tilde{I}}_{K}$, ${\tilde{I}}_{\mathrm{Na}}$, and ${\tilde{I}}_{\mathrm{Ca}}$ are the sodium, potassium, and calcium currents in the frequency domain, respectively.

#### 3.2.3. Thermal Noise

Thermal noise is known as thermal agitation which stems from the random movement of electrical charges in the electrical systems. Thermal noise has a significant impact on the performance of the receiving cardiomyocyte [42]. The considered cardiomyocyte communication system suggested by us consists of different passive components, i.e., cytosol-related resistors generated by intracellular dynamics, and membrane-related resistors and capacitors generated by the phospholipid bilayer cell-membrane. However, as described in [37], we ignore thermal noise evoked by the cytosol-related resistors, and only consider membrane-related passive components to calculate the PSD of the thermal noise

(17) $$\begin{array}{c} {\tilde{S}}_{N_{2}}^{3}\left(jf\right)=\frac{2kT}{ℜ\{z_{m}\left(jf\right)\}}, \end{array}$$

where *k* is Boltzmann constant and *T* is the absolute temperature.

Ultimately, the overall effect of the membrane-related noise is now described as

(18) $$\begin{array}{c} {\tilde{S}}_{N2_{u}}\left(jf\right)={\tilde{S}}_{N_{2}}^{1}\left(jf\right)+{\tilde{S}}_{N_{2}}^{2}\left(jf\right)+{\tilde{S}}_{N_{2}}^{3}\left(jf\right), \end{array}$$

or, alternatively,

(19) $$\begin{array}{c} {\tilde{S}}_{N_{2}}\left(x,jf\right)=\int_{0}^{x}{\tilde{S}}_{N2_{u}}\left(jf\right)|Z\left(y,jf\right){|}^{2}dy, \end{array}$$

where Z(y,jf) is given in (7) and ${\tilde{S}}_{N2_{u}}\left(jf\right)$ in (18).

### 3.3. Noisy Input-Output Relation

For the linear cardiomyocyte communication system, the PSD of the signal received at the receiving cardiomyocyte ${\tilde{S}}_{Rx}\left(x,f\right)$ is

(20) $$\begin{array}{ccc} {\tilde{S}}_{Rx}\left(x,jf\right) & = & |Z\left(x,jf\right){|}^{2}{\tilde{S}}_{Tx}\left(jf\right)+{\tilde{S}}_{N_{2}}\left(x,jf\right)\\ & = & \left(S_{Tx}\left(jf\right)+S_{N_{1}}\left(jf\right)\right)|Z\left(x,jf\right){|}^{2}+{\tilde{S}}_{N_{2}}\left(x,jf\right), \end{array}$$

where ${\tilde{S}}_{Tx}\left(jf\right)$ is given in (13), ${\tilde{S}}_{N_{2}}\left(x,jf\right)$ in (19) and Z(x,jf) in (7).

## 4. Numerical Simulations

Analysis of the performance of the subthreshold cardiac communication system relies on the computational action potential models. A general and uniform action potential model for cardiomyocytes generally does not exist because the ionic current components vary for different cell types. The verification of the model depends on the experimental data. In this study, we linearize the membrane into the primary circuit and study the subthreshold cardiomyocyte communication by using the Luo-Rudy model, which is based on the Hodgkin-Huxley-type formalism [43,44]. The Luo-Rudy model is one of the commonly used *ventricular cardiomyocyte* action potential models and considers the most typical ionic current components, particularly, the sodium channel function operating in the subthreshold regime. The Luo-Rudy model provides the coefficients of different ionic channels. We list the general parameters used in our simulation framework in Table 2. As of note, the channel density and conductance of single sodium and calcium ionic channels are taken from the Reuter et al.’s experimental work [45,46,47], whereas the channel density and conductance of single potassium ionic channels are taken from the Shibasaki’s experimental work [48]. Other parameters mainly originate from the Luo-Rudy model related works [27,43].

The linearization depends on the holding potential, which can be any value in the subthreshold range. In the simulation framework, we set the holding potential to be equal to the resting potential of −84 mV since it enables a broader amplitude range for data transmission: the stimulation range is 24 mV when the threshold is −60 mV. Table 3 lists the circuit parameters obtained by applying the method in Appendix A.1 to linearize the membrane at the selected holding potential; the parameters used as an input to the linearization method are from Table 2. The linearized values of phenomenological resistors and inductors of sodium, potassium, and calcium are negative as the holding potential is smaller than their reversal potentials [26].

**Figure 6:**
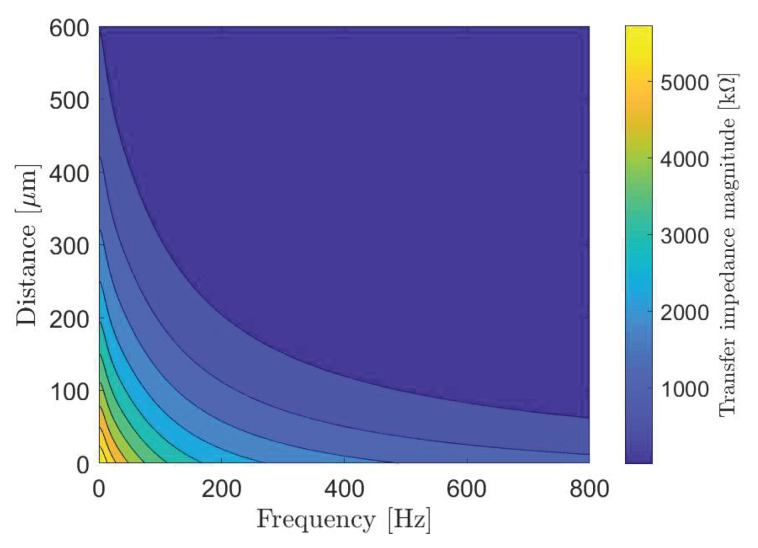
The transfer impedance of the subthreshold cardiac communication system.

Figure 6 shows changes in the transfer impedance, that we compute from (7), against the system frequency and propagation distance between the transmitting nano-actuator and the receiving nano-actuator. As intuitively expected, the transfer impedance decreases with an increase in both frequency and distance. According to the peak transfer impedance value and given action potential threshold, we determine the maximum stimulation current of 3.81 nA which can be applied to the cells in the data transmission mode of the cardiac system.

**Figure 7:**
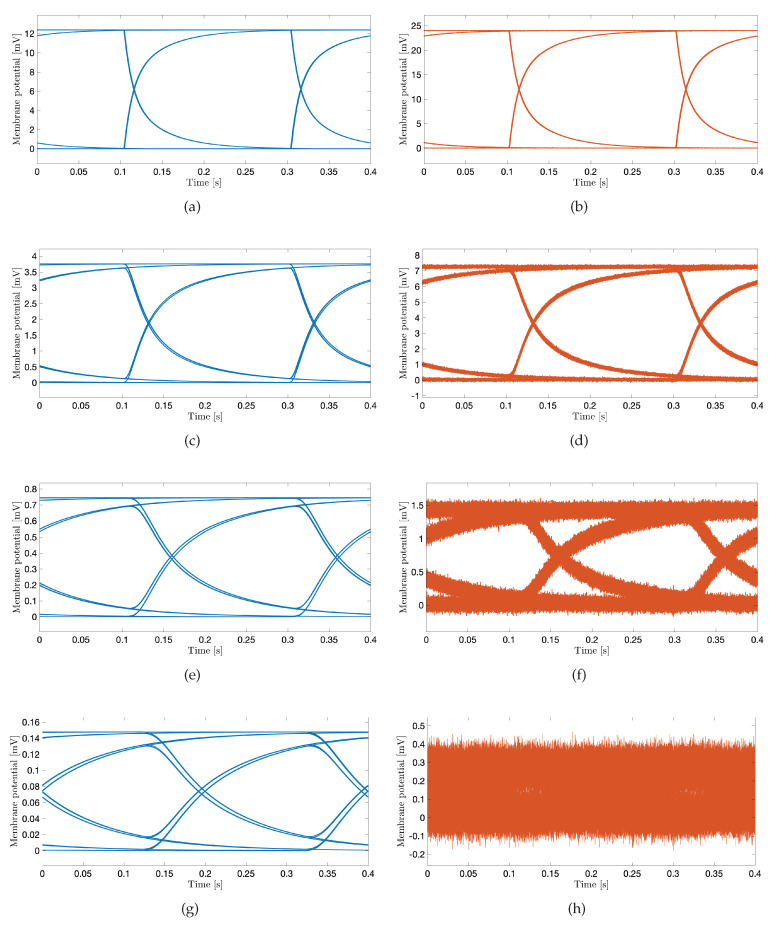
The eye diagram of subthreshold cardiac communication system. The stimulation amplitude is 3 nA, and the transmission rate is 5 bit/s. Blue curves correspond to noiseless scenarios; orange curves correspond to noisy scenarios. (**a**,**b**): The transmission distance corresponds to the one-cell length. (**c**,**d**): The transmission distance corresponds to the four-cell length. (**e**,**f**): The transmission distance corresponds to the eight-cell length. (**g**,**h**): The transmission distance corresponds to the twelve-cell length.

Selecting proper transmission rates in time bins intended for data transmission (Figure 2) is important to minimize intersymbol interference at the receiver point. For the system performance demonstration, we select the bit transmission rate of 5 bit/s to plot eye diagrams in Figure 7 considering different system parameters. An eye diagram is a tool used in communications engineering for the evaluation of the combined effects of inter-symbol interference and channel noise on the performance of a baseband signal-transmission system. An open eye diagram corresponds to minimal signal distortion. A closed eye diagram corresponds to signal distortion. We plot eye diagrams at different distances considering the noiseless and noisy scenarios, respectively. We observe that in short-distance transmission systems the eye openings are wide with plentiful margin decisions at the receiver regardless of the effect of noise (Figure 7a–d). As expected, however, the effect of noise combined with the increased distance among nano-actuators appears as the closure of the eye diagram with low-amplitude signals (Figure 7f,h). In such scenarios, highly sensitive receivers are required by the system to measure the low-amplitude signals. Of note, present high-performance microelectrodes could ideally measure potentials as small as 0.015 mV [49]. The completely closed eye appears in a noisy considered scenario where twelve cells compose the communication channel (Figure 7h).

**Figure 8:**
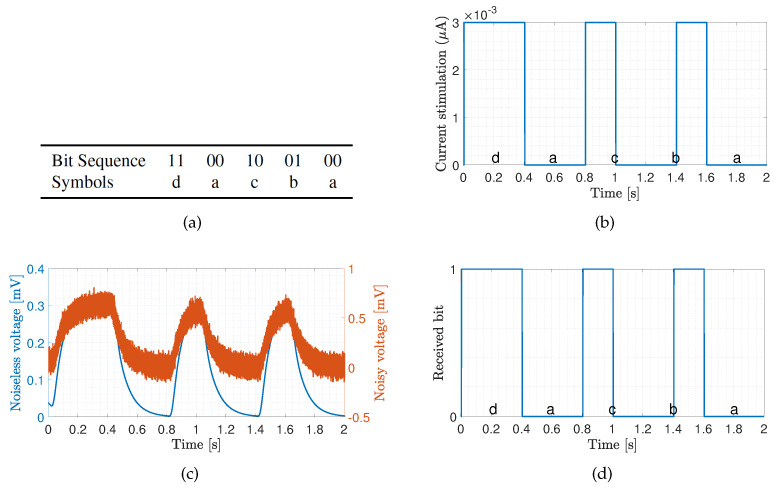
Transmission of bit sequence over ten cardiomyocytes with stimulation signal amplitude of 3 nA. The transmission bin of 2 seconds has been selected to demonstrate the system performance. (**a**) Sample bit sequence and associated symbols. (**b**) Transmitted signal. (**c**) Received signal. (**d**) Decoded signal.

**Figure 9:**
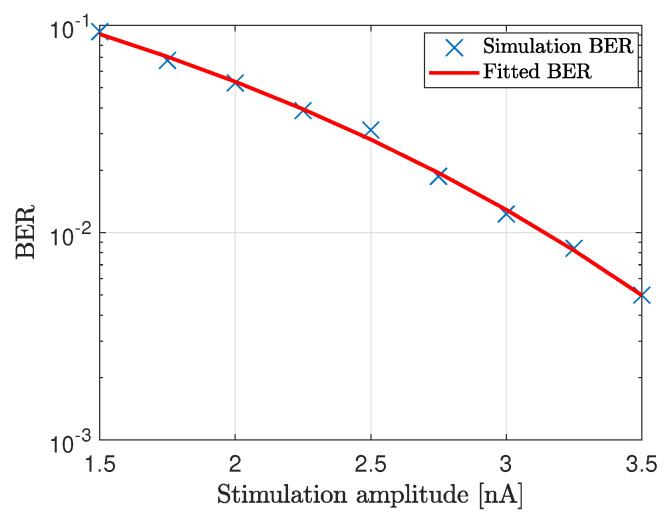
BER of the communication system where ten cardiomyocytes form the propagating channel.

We now demonstrate coded data transmission over the channel composed of ten cardiomyocytes using Amplitude-Shift Keying. This modulation technique implies that binary 1 is represented by transmitting a fixed-amplitude wave for a fixed time duration; otherwise, binary 0 is represented. We select n=2 bits to represent a symbol, meaning that $M=2^{n}=4$ different symbols could be encoded, i.e., symbols ‘a’, ‘b’, ‘c’ and ‘d’ represented by 00, 01, 10 and 11, respectively. We randomly generate 1000 bits to ensure that the symbols are equally represented. The probabilities of the symbols ‘a’, ‘b’, ‘c’ and ‘d’ are 0.240, 0.254, 0.250 and 0.256, respectively. Figure 8a,b show a small portion of the transmission bit stream consisting of 10 bits. Figure 8c shows a small portion of the received bit stream consisting of 10 bits without and with the consideration of noise sources, respectively. We observe the positive effect of the noise when bit 1 is transmitted. This effect stems from the input-dependent noise that apparently enhances the cardiomyocyte stimulation. Conversely, we observe the negative effect of the noise when bit 0 is transmitted. This effect stems from the channel noise that decreases the margin decision. Nonetheless, with the proper threshold selected according to the eye diagram, the receiver can successfully decode the received signals to corresponding 1/0. As shown in Figure 8d, the receiver decodes the signal successfully in both scenarios. As of note, the performance is highly dependent on the stimulation amplitude. Based on randomly generated and transmitted 10,000 bits, we evaluate the bit error rate (BER) when changing the stimulation amplitude starting from 1.5 nA, as shown in Figure 9. As expected, the BER decreases with the stimulation amplitude and reaches the minimum value of 5 × 10${}^{-3}$ when the stimulation amplitude is 3.5 nA.

## 5. Concluding Remarks

We considered the communication system between transmitting and receiving nano-actuators within a multi-nodal pacemaker network. The subthreshold cardiac communication paradigm considered in this paper offers a potentially groundbreaking method for data transmission within the heart. The demonstrated transmissions showed that data could be successfully transmitted in the subthreshold domain over tens of cells only. The results are still insightful and provide initial information on how to distribute and deploy the relay nano-actuating node(s) in the multi-nodal pacemaker network. Combining the subthreshold cardiac communication system with the optimal stimulation methods may provide an energy-efficient pacing of cardiomyocytes.

The time bins when transmission can happen correspond to the duration of the ventricular diastole phase which is approximately 430 ms [50]. Based on the presented results and analyzed bit rates, a very limited amount of data could be transmitted. Nonetheless, for the essential function of a multi-nodal leadless pacemaker, where the nano-actuators primarily sense membrane potentials of the corresponding cells and assist in pacing, the proposed communication system could enable transmission of a status of the node’s stimulation activity ensuring coordinated operation within the network.

The numerical results presented in the paper are based on the computational Luo-Rudy model of cardiac action potentials whose parameters are used to linearize the cardiac circuit. Though the Luo-Rudy model is not perfect, e.g., it does not consider the stochasticity of single ionic channels, it still serves as the basis for most computational models and studies involving myocytes and provides the coefficients for different ionic channels. For more precise results, in-vitro experiments are needed to obtain precise parameters for the specific cells. The experiments will also reveal the dynamics of the gap junction resistances as they change according to the potential between the gap junctions. In this study, we considered the resistance of the gap as constant.

Action potentials may affect the performance of the subthreshold transmission due to variations (jitter) of their initial times in different physiological environments. The action potential duration also varies in different physiological environments, which directly affects the length of the temporal bins intended for data transmission. On the contrary, prolonged data transmission in time bins between consecutive action potentials may affect action potentials. Besides, when multiple nano-actuators transmit data at the same time, the interference to the EGM (electrogram) and ECG may affect the performance of the proposed system. The EGM is used to measure the local signal in the tissue level. To measure the interference of encoding signals to the EGM, we would need to (1) apply more advanced 3D topological tissue models, (2) analyze the coupling between cells, (3) identify possible multiple paths between the transmitting and receiving nano-actuators, and, ultimately, (4) consider the position and timing/synchronization issue of nano-actuators. This indicates the direction for future work in this field.

Furthermore, future work should include the complex structure of the cardiomyocytes, such as syncytium structure or network structure, together with the timing of signal transmission between nano-actuators and the gateway/hub. Ultimately, in-vitro and in-vivo experiments are urgently needed to generate more precise circuit models and obtain real data on subthreshold membrane potentials propagation which will be used to verify the numerical results presented in this paper.

## Figures and Tables

**Table 1:** Defined symbols used throughout the paper.

Parameter	Description	Unit
$S_{Tx}\left(jf\right)$	Input current PSD	μA${}^{2}$/Hz
$z_{m}\left(jf\right)$	Membrane impedance (per unit length)	kΩ· cm
$Z_{m}\left(jf\right)$	Resistivity of membrane	kΩ· cm${}^{2}$
$z_{l}$	Equivalent longitudinal impedance (per unit length)	kΩ/cm
$Z_{l}$	Equivalent longitudinal resistivity	kΩ· cm
Z(x,jf)	Transfer impedance	kΩ
$S_{Rx}\left(jf\right)$	Output voltage PSD	mV${}^{2}$/Hz
$i_{Tx}\left(t\right)$	Input current	μA
${\tilde{i}}_{Tx}\left(t\right)$	Input current corrupted with input-dependent noise	μA
$i_{N_{1}}\left(t\right)$	Input-dependent noise current	μA
$S_{N_{1}}\left(jf\right)$	Current PSD of input-dependent noise current	μA${}^{2}$/Hz
${\tilde{S}}_{Tx}\left(jf\right)$	Current PSD of the input corrupted with input-dependent noise	μA${}^{2}$/Hz
${\tilde{S}}_{N_{2}}^{1}\left(jf\right)$	Current PSD of voltage-gated channel noise	μA${}^{2}$/Hz/cm
${\tilde{S}}_{N_{2}}^{2}\left(jf\right)$	Current PSD of shot noise	μA${}^{2}$/Hz/cm
${\tilde{S}}_{N_{2}}^{3}\left(jf\right)$	Current PSD of thermal noise	μA${}^{2}$/Hz/cm
${\tilde{S}}_{K}\left(jf\right)$	Current PSD of potassium ions	μA${}^{2}$/Hz/cm
${\tilde{S}}_{\mathrm{Na}}\left(jf\right)$	Current PSD of sodium ions	μA${}^{2}$/Hz/cm
${\tilde{S}}_{\mathrm{Ca}}\left(jf\right)$	Current PSD of calcium ions	μA${}^{2}$/Hz/cm
${\tilde{S}}_{N2_{u}}\left(jf\right)$	Current PSD of membrane-related noise	μA${}^{2}$/Hz/cm
${\tilde{S}}_{N_{2}}\left(x,jf\right)$	Voltage PSD of membrane-related noise	mV${}^{2}$/Hz
${\tilde{S}}_{Rx}\left(x,jf\right)$	Output noisy voltage PSD	mV${}^{2}$/Hz

**Table 2:** Parameters used in the simulation framework.

Parameter	Description	Value	Unit
$C_{m}$	Specific membrane capacitance	1	μF/cm${}^{2}$
$E_{\mathrm{Na}}$	Reversal potential	54.4	mV
$E_{K}$	Reversal potential	−77	mV
$E_{si}$	Reversal potential	40	mV
$\gamma_{{\mathrm{Na}}^{+}}$	Channel conductance	15	pS
$\eta_{\mathrm{Na}}^{patch}$	Channel density	1–16	/μm${}^{2}$
$\gamma_{\mathrm{Ca}}$	Channel conductance	9–25	pS
$\eta_{\mathrm{Ca}}^{patch}$	Channel density	0.5–5	/μm${}^{2}$
$\gamma_{K^{+}}$	Channel conductance	1.6	pS
$\eta_{K}^{patch}$	Channel density	0.7	/μm${}^{2}$
$Z_{l}$	Equivalent longitudinal resistivity	600	Ω·cm
$S_{V}$	Surface-to-volume ratio	4440	cm${}^{-1}$
$L_{cell}$	Cell length	100	μm
*a*	Cell radius	10	μm

**Table 3:** Parameters used for membrane linearization.

Parameter	Value	Unit
$R_{\mathrm{Na}}$	1.13 × 10${}^{7}$	kΩ·cm${}^{2}$
$r_{m}$	−1.64 × 10${}^{5}$	kΩ·cm${}^{2}$
$L_{m}$	−0.98	kΩ·s·cm${}^{2}$
$r_{h}$	1.92 × 10${}^{7}$	kΩ·cm${}^{2}$
$L_{h}$	7.74 × 10${}^{4}$	kΩ·s·cm${}^{2}$
$r_{j}$	3.13 × 10${}^{7}$	kΩ·cm${}^{2}$
$L_{j}$	5.32 × 10${}^{5}$	kΩ·s·cm${}^{2}$
$R_{K}$	2.13 × 10${}^{3}$	kΩ·cm${}^{2}$
$r_{X}$	−3.11 × 10${}^{3}$	kΩ·cm${}^{2}$
$L_{X}$	−711.64	kΩ·s·cm${}^{2}$
$R_{X_{i}}$	2.32 × 10${}^{6}$	kΩ·cm${}^{2}$
$R_{\mathrm{Ca}}$	160.35	kΩ·cm${}^{2}$
$r_{d}$	−15.29	kΩ·cm${}^{2}$
$L_{d}$	−0.13	kΩ·s·cm${}^{2}$
$r_{f}$	3.31 × 10${}^{5}$	kΩ·cm${}^{2}$
$L_{f}$	1.76 × 10${}^{4}$	kΩ·s·cm${}^{2}$
$R_{o}$	6.11 × 10${}^{8}$	kΩ·cm${}^{2}$

## Appendix A

### Appendix A.1. Membrane Linearization

We linearize the sodium-, potassium- and calcium currents in cardiomyocytes following the same methodology presented in [25]. The sodium current is expressed as [43]

(A1) $$\begin{array}{ccc} I_{\mathrm{Na}} & = & {\bar{g}}_{\mathrm{Na}}^{patch}m^{3}hj\left(v-E_{\mathrm{Na}}\right), \end{array}$$

(A2) $$\begin{array}{ccc} {\bar{g}}_{\mathrm{Na}}^{patch} & = & \eta_{\mathrm{Na}}^{patch}\gamma_{\mathrm{Na}}, \end{array}$$

where ${\bar{g}}_{\mathrm{Na}}^{patch}$ is the maximum conductance of sodium channels, *v* is the membrane potential, $E_{\mathrm{Na}}$ is the reversal potential, $\eta_{\mathrm{Na}}^{patch}$ is the sodium channel density on unit area and it is 10 in the simulation framework, and $\gamma_{\mathrm{Na}}$ is the conductance of single sodium channel. *m*, *h* and *j* represent the sodium active channel parameter, the sodium inactive channel parameter, and the sodium slow inactive channel parameter, respectively. They are functions of time and membrane potential and indicate the subunits of the sodium channel.

The membrane potential can be linearized around small changes denoted with δ. Accordingly, the sodium current variation can be expressed as

(A3) $$\begin{array}{ccc} \delta I_{\mathrm{Na}} & = & \delta v[{\bar{g}}_{\mathrm{Na}}^{patch}m_{v}^{3}h_{v}j_{v}+3{\bar{g}}_{\mathrm{Na}}^{patch}m_{v}^{2}h_{v}j_{v}\left(v-E_{\mathrm{Na}}\right)\delta m+\\ & & {\bar{g}}_{\mathrm{Na}}^{patch}m_{v}^{3}j_{v}\left(v-E_{\mathrm{Na}}\right)\delta h+{\bar{g}}_{\mathrm{Na}}^{patch}m_{v}^{3}h_{v}\left(v-E_{\mathrm{Na}}\right)\delta j]\\ & = & \delta I_{\mathrm{Na}1}+\delta I_{\mathrm{Na}2}+\delta I_{\mathrm{Na}3}+\delta I_{\mathrm{Na}4}, \end{array}$$

where $m_{v}$, $h_{v}$ and $j_{v}$ are the parameters at steady-state. In (A3), $\delta I_{\mathrm{Na}1}$ is constant because ${\bar{g}}_{\mathrm{Na}}$, $m_{v}$, $h_{v}$ and $j_{v}$ are constant in the steady state. $\delta I_{\mathrm{Na}2}$, $\delta I_{\mathrm{Na}3}$ and $\delta I_{\mathrm{Na}4}$ indicate the small current changes of the subunit *m*, *h*, *j*, respectively. We consider m variation in the following and extend results to *h* and *j*.

The active subunit *m* has two states: open and closed. *m* changes with time as

(A4) $$\begin{array}{c} \frac{dm}{dt}=\alpha_{m}\left(1-m\right)-\beta_{m}m, \end{array}$$

where $\alpha_{m}$ and $\beta_{m}$ are the coefficients of the subunit from closing state to opening state and opening state to closing state, respectively. $\alpha_{m}$ and $\beta_{m}$ are functions of *v* that change for a small variation as

(A5) $$\begin{array}{ccc} \frac{\delta \alpha_{m}}{dt} & = & {\left(\frac{d\alpha_{m}}{dv}\right)}_{v}\delta v, \end{array}$$

(A6) $$\begin{array}{ccc} \frac{\delta \beta_{m}}{dt} & = & {\left(\frac{d\beta_{m}}{dv}\right)}_{v}\delta v. \end{array}$$

We then yield

(A7) $$\begin{array}{ccc} \frac{d\delta_{m}}{dt} & = & {\left(\frac{d\alpha_{m}}{dv}\right)}_{v}\delta v-\left(\alpha_{m}+\beta_{m}\right)\delta m-\\ & & m_{v}{\left(\frac{d\alpha_{m}}{dv}\right)}_{v}\delta v-m_{v}{\left(\frac{d\beta_{m}}{dv}\right)}_{v}\delta v \end{array}$$

or

(A8) $$\begin{array}{c} \left(p+\alpha_{m}+\beta_{m}\right)\delta_{m}\\ =\left[{\left(\frac{d\alpha_{m}}{dv}\right)}_{v}-m_{v}{\left(\frac{d(\alpha_{m}+\beta_{m})}{dv}\right)}_{v}\right]\delta v, \end{array}$$

where p≡d/dt. Finally, we yield

(A9) $$\begin{array}{ccc} \delta I_{\mathrm{Na}2} & = & 3{\bar{g}}_{\mathrm{Na}}^{patch}m_{v}^{2}h_{v}j_{v}\left(v-E_{\mathrm{Na}}\right)\delta m\delta v\\ & = & 3{\bar{g}}_{\mathrm{Na}}^{patch}m_{v}^{2}h_{v}j_{v}\left(v-E_{\mathrm{Na}}\right)\frac{{\left(\frac{d\alpha_{m}}{dv}\right)}_{v}-m_{v}{\left(\frac{d(\alpha_{m}+\beta_{m})}{dv}\right)}_{v}}{p+\alpha_{m}+\beta_{m}}\delta v\\ & = & \frac{a}{p+b}, \end{array}$$

where $a\equiv 3{\bar{g}}_{\mathrm{Na}}^{patch}m_{v}^{2}h_{v}j_{v}\left(v-E_{\mathrm{Na}}\right)\left[{\left(\frac{d\alpha_{m}}{dv}\right)}_{v}-m_{v}{\left(\frac{d(\alpha_{m}+\beta_{m})}{dv}\right)}_{v}\right]$ and $b\equiv \alpha_{m}+\beta_{m}$.

For $\delta I_{\mathrm{Na}1}$, we yield

(A10) $$\begin{array}{c} \frac{\delta v}{\delta I_{\mathrm{Na}1}}=\frac{1}{{\bar{g}}_{\mathrm{Na}}^{patch}m_{v}^{3}h_{v}j_{v}}, \end{array}$$

which we abstract as a resistor generated by sodium channels

(A11) $$\begin{array}{c} R_{\mathrm{Na}}=\frac{1}{{\bar{g}}_{\mathrm{Na}}^{patch}m_{v}^{3}h_{v}j_{v}}. \end{array}$$

For $\delta I_{\mathrm{Na}2}$ and according to (A8), we yield

(A12) $$\begin{array}{c} \frac{\delta v}{\delta I_{\mathrm{Na}2}}=\frac{1}{a}p+\frac{b}{a}, \end{array}$$

which we abstract as a resistor $r_{m}$ in serial connection with an inductor $L_{m}$

(A13) $$\begin{array}{ccc} r_{m} & = & \frac{b}{a}, \end{array}$$

(A14) $$\begin{array}{ccc} L_{m} & = & \frac{1}{a}=\frac{r_{m}}{\alpha_{m}+\beta_{m}}. \end{array}$$

Similar to $\delta I_{\mathrm{Na}2}$, we define resistor and inductor parameters for $\delta I_{\mathrm{Na}3}$ and $\delta I_{\mathrm{Na}4}$.

### Appendix A.2. Derivation of Current Noise PSD

Similar to neuronal ionic channels, the sodium, potassium, and calcium channels in cardiomyocytes can be considered as finite-state Markov chains. Considering the sodium channels, the autocovariance of the corresponding current is derived from [38,51,52]

(A15) $$\begin{array}{c} C_{I\mathrm{Na}}\left(t\right)=\eta_{\mathrm{Na}}^{line}\gamma_{\mathrm{Na}}^{2}{\left(v-E_{\mathrm{Na}}\right)}^{2}\left(m_{v}^{3}h_{v}j_{v}P_{\mathrm{Na},0|0}\left(t\right)-{\left(m_{v}^{3}h_{v}j_{v}\right)}^{2}\right), \end{array}$$

where $\eta_{\mathrm{Na}}^{line}=2\pi a\eta_{\mathrm{Na}}^{patch}$ is the sodium channel density in unit length, $\gamma_{\mathrm{Na}}$ is the conductance of a single channel, and $m_{v}$, $h_{v}$, and $j_{v}$ are steady-state values when the membrane potential is *v*. $P_{\mathrm{Na},0|0}\left(t\right)$ is the conditional probability of all the subunits of sodium channels to open at time t=0

(A16) $$\begin{array}{ccc} P_{\mathrm{Na},0|0}\left(t\right) & = & {\left(m_{v}+\left(1-m_{v}\right)e^{-t/\tau_{m}}\right)}^{3}\left(h_{v}+\left(1-h_{v}\right)e^{-t/\tau_{h}}\right)\left(j_{v}+\left(1-j_{v}\right)e^{-t/\tau_{j}}\right), \end{array}$$

where $\tau_{m}$, $\tau_{h}$ and $\tau_{j}$ are the time constant of *m*, *h* and *j*, respectively.

By using the Wiener-Khinchine theorem, the current PSD is

(A17) $$\begin{array}{c} S_{I\mathrm{Na}}\left(jf\right)=\int_{-\infty }^{\infty }C_{I\mathrm{Na}}\left(t\right)e^{-j2\pi ft}dt\\ =6Am_{v}^{2}h_{v}j_{v}\left(1-m_{v}\right)\tau_{m}\frac{1}{1+{\left(2\pi f\tau_{m}\right)}^{2}}+\\ 6Am_{v}h_{v}j_{v}{\left(1-m_{v}\right)}^{2}\frac{\tau_{m}}{2}\frac{1}{1+{\left(\frac{\tau_{m}}{2}2\pi f\right)}^{2}}+\\ 2Ah_{v}j_{v}{\left(1-m_{v}\right)}^{3}\frac{\tau_{m}}{3}\frac{1}{1+{\left(\frac{\tau_{m}}{3}2\pi f\right)}^{2}}+\\ 6Am_{v}^{2}h_{v}\left(1-j_{v}\right)\left(1-m_{v}\right)\frac{\tau_{m}\tau_{j}}{\tau_{m}+\tau_{j}}\frac{1}{1+{\left(\frac{\tau_{m}\tau_{j}}{\tau_{m}+\tau_{j}}2\pi f\right)}^{2}}+\\ 6Am_{v}{\left(1-m_{v}\right)}^{2}h_{v}\left(1-j_{v}\right)\frac{2\tau_{m}\tau_{j}}{\tau_{m}+2\tau_{j}}\frac{1}{1+{\left(\frac{2\tau_{m}\tau_{j}}{\tau_{m}+2\tau_{j}}2\pi f\right)}^{2}}+\\ 2A{\left(1-m_{v}\right)}^{3}h_{v}\left(1-j_{v}\right)\frac{3\tau_{m}\tau_{j}}{\tau_{m}+3\tau_{j}}\frac{1}{1+{\left(\frac{3\tau_{m}\tau_{j}}{\tau_{m}+3\tau_{j}}2\pi f\right)}^{2}}+\\ 6Am_{v}^{2}\left(1-m_{v}\right)\left(1-h_{v}\right)j_{v}\frac{\tau_{m}\tau_{h}}{\tau_{m}+\tau_{h}}\frac{1}{1+{\left(\frac{\tau_{m}\tau_{h}}{\tau_{m}+\tau_{h}}2\pi f\right)}^{2}}+\\ 6Am_{v}{\left(1-m_{v}\right)}^{2}\left(1-h_{v}\right)j_{v}\frac{2\tau_{m}\tau_{h}}{\tau_{m}+2\tau_{h}}\frac{1}{1+{\left(\frac{2\tau_{m}\tau_{h}}{\tau_{m}+2\tau_{h}}2\pi f\right)}^{2}}+\\ 2A{\left(1-m_{v}\right)}^{3}\left(1-h_{v}\right)j_{v}\frac{3\tau_{m}\tau_{h}}{\tau_{m}+3\tau_{h}}\frac{1}{1+{\left(\frac{3\tau_{m}\tau_{h}}{\tau_{m}+3\tau_{h}}2\pi f\right)}^{2}}+\\ 6Am_{v}^{2}\left(1-m_{v}\right)\left(1-h_{v}\right)\left(1-j_{v}\right)\frac{\tau_{m}\tau_{h}\tau_{j}}{\tau_{h}\tau_{j}+\tau_{m}\tau_{j}+\tau_{m}\tau_{h}}\frac{1}{1+{\left(\frac{\tau_{m}\tau_{h}\tau_{j}}{\tau_{h}\tau_{j}+\tau_{m}\tau_{j}+\tau_{m}\tau_{h}}2\pi f\right)}^{2}}+\\ 6Am_{v}^{3-2}{\left(1-m_{v}\right)}^{2}\left(1-h_{v}\right)\left(1-j_{v}\right)\frac{\tau_{m}\tau_{h}\tau_{j}}{2\tau_{h}\tau_{j}+\tau_{m}\tau_{j}+\tau_{m}\tau_{h}}\frac{1}{1+{\left(\frac{\tau_{m}\tau_{h}\tau_{j}}{2\tau_{h}\tau_{j}+\tau_{m}\tau_{j}+\tau_{m}\tau_{h}}2\pi f\right)}^{2}}+\\ 2A{\left(1-m_{v}\right)}^{3}\left(1-h_{v}\right)\left(1-j_{v}\right)\frac{\tau_{m}\tau_{h}\tau_{j}}{3\tau_{h}\tau_{j}+\tau_{m}\tau_{j}+\tau_{m}\tau_{h}}\frac{1}{1+{\left(\frac{\tau_{m}\tau_{h}\tau_{j}}{3\tau_{h}\tau_{j}+\tau_{m}\tau_{j}+\tau_{m}\tau_{h}}2\pi f\right)}^{2}}+\\ 2Am_{v}^{3}\left(1-h_{v}\right)\left(1-j_{v}\right)\frac{\tau_{h}\tau_{j}}{\tau_{h}+\tau_{j}}\frac{1}{1+{\left(\frac{\tau_{h}\tau_{j}}{\tau_{h}+\tau_{j}}2\pi f\right)}^{2}}+\\ 2Am_{v}^{3}\left(1-h_{v}\right)j_{v}\tau_{h}\frac{1}{1+{\left(\tau_{h}2\pi f\right)}^{2}}+\\ 2Am_{v}^{3}h_{v}\left(1-j_{v}\right)\tau_{j}\frac{1}{1+{\left(\tau_{j}2\pi f\right)}^{2}}, \end{array}$$

where $A=\eta_{\mathrm{Na}}^{line}\gamma_{\mathrm{Na}}^{2}{\left(v-E_{\mathrm{Na}}\right)}^{2}m_{v}^{3}h_{v}j_{v}.$

Similar to sodium, the autocovariance of the calcium current is

(A18) $$\begin{array}{c} C_{\mathrm{Ca}}\left(t\right)=\eta_{\mathrm{Ca}}^{line}\gamma_{\mathrm{Ca}}^{2}{\left(v-E_{si}\right)}^{2}d_{v}f_{v}\left(P_{\mathrm{Ca},0|0}\left(t\right)-d_{v}f_{v}\right), \end{array}$$

where $\eta_{\mathrm{Ca}}^{line}=2\pi a\eta_{\mathrm{Ca}}^{patch}$ is the calcium channel density in unit length, $\gamma_{\mathrm{Ca}}$ is the conductance of a single channel, $E_{si}$ is the reversal potential of calcium, and $d_{v}$ and $f_{v}$ are steady-state values when the membrane potential is *v*. $P_{\mathrm{Ca},0|0}\left(t\right)$ is the conditional probability of all the subunits of calcium channels to open at time t=0

$$\begin{array}{c} P_{\mathrm{Ca},0|0}\left(t\right)=\left(d_{v}+\left(1-d_{v}\right)e^{-t/\tau_{d}}\right)\left(f_{v}+\left(1-f_{v}\right)e^{-t/\tau_{f}}\right), \end{array}$$

where $\tau_{d}$ and $\tau_{f}$ are the time constant of *d* and *f*, respectively. The current PSD is

(A19) $$\begin{array}{ccc} S_{I\mathrm{Ca}}\left(jf\right) & = & \int_{-\infty }^{\infty }C_{\mathrm{Ca}}\left(t\right)e^{-j2\pi ft}dt. \end{array}$$

The autocovariance of the potassium current is

(A20) $$\begin{array}{c} C_{IK}\left(t\right)=\eta_{K}^{line}\gamma_{K}^{2}\left(v-E_{K}\right)X_{v}\left(P_{K,0|0}\left(t\right)-X_{v}\right), \end{array}$$

where $\eta_{K}^{line}=2\pi a\eta_{K}^{patch}$ is the potassium channel density in unit length, $\gamma_{K}$ is the conductance of a single channel, $E_{K}$ is the reversal potential of potassium, and $X_{v}$ is steady-state values when the membrane potential is *v*. $P_{K,0|0}\left(t\right)$ is the conditional probability of the potassium activation channels to open at time t=0

(A21) $$P_{K,0|0}\left(t\right)=X_{v}+\left(1-X_{v}\right)e^{-t/\tau_{X}},$$

where $\tau_{X}$ is the time constant of *X*. The current PSD is

(A22) $$\begin{array}{ccc} S_{IK}\left(jf\right) & = & \int_{-\infty }^{\infty }C_{IK}\left(t\right)e^{-j2\pi ft}dt. \end{array}$$
